# A novel method for the collection of nanoscopic vesicles from an organotypic culture model

**DOI:** 10.1039/c7ra12511a

**Published:** 2018-02-16

**Authors:** Alexandra Iordachescu, Philippa Hulley, Liam M. Grover

**Affiliations:** School of Chemical Engineering, University of Birmingham Edgbaston Birmingham B15 2TT UK a.iordachescu@bham.ac.uk; Botnar Research Centre, University of Oxford Old Road, Headington Oxford OX3 7LD UK

## Abstract

Nanovesicles, exosomes and other membrane bound particles excreted by cells are currently gaining research attention since they have been shown to play a significant role in many biologically related processes. Vesicles are now thought to mediate cellular communication, transmission of some diseases and pathologically mediated calcification. Matrix vesicles have long been proposed to be central to the controlled mineralisation of bone. They remain relatively poorly studied, however, since they are challenging to extract from biological media. One difficulty is the presence of a mineral content in comparison to pure lipid vesicles, meaning that standard separation process such as ultracentrifugation are unable to precisely separate on the basis of size or weight. In this paper we report the separation of matrix vesicles from an organotypic bone culture system using a process of immunoprecipitation. Matrix vesicles were extracted using polymeric beads that were modified with an antibody for tissue non-specific alkaline phosphatase (TNALP), a surface marker abundant in bone-derived vesicles. The vesicles isolated were positive for adenosine triphosphate (ATP), the substrate for TNALP and were demonstrated to have a high-binding affinity to type I collagen, the principal collagen type found in bone. This protocol enables more detailed study of the process and regulation of mineralisation.

## Abstract 2

Cellular nanovesicles have been shown to play a significant role in many biological processes. Organotypic bone culture systems are a source of physiologically-relevant mineralisation vesicles, which can be immuno-selected for investigation.

## Graphical abstract

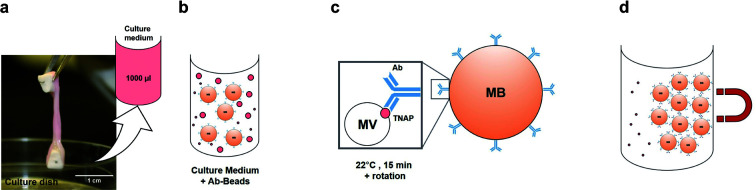

## Introduction

The process of ossification takes places through a cell-mediated route, where cartilaginous matrix built by chondrocytic and osteoblastic cells becomes mineralised in an organised manner, ultimately giving rise to the mature bone tissue.^1^ It is generally accepted that the process of calcium and phosphate deposition in cartilage, bone and dentin is mediated by exosome/vesicle-like nano-structures, generally referred to as matrix vesicles (MVs).^2–4^ These nanovesicles are thought to bud off from the plasma membrane of hypertrophic chondrocytes and osteoblasts^5^ and contain a combination of compounds which allow the localised deposition of hydroxyapatite (or a poorly crystalline apatite), which ultimately becomes precipitated on the surface of collagen fibrils,^2,6^ forming nucleation points^7^ for further mineral growth.

The selection of specific vesicles present in the environment of cells which are actively involved in building the bone matrix (*i.e.* osteoblasts and osteocytes) can be useful for further understanding the ossification process during both physiological and aberrant states.

This has been an area of intense research in the past years and numerous methods have become available for selecting these nanostructures for further characterisation, each presenting various advantages and disadvantages.^8^ Traditional protocols for selecting these small vesicles involve ultracentrifugation (UC),^9^ which allows the separation of matrix vesicles on the basis that larger particles sediment faster, while the smaller particles remain in the supernatant and can be recovered using further centrifugation steps. In the case of matrix vesicles, the mineral phase contained by the vesicles increases their weight such that they pellet at a faster rate.^9^ Whilst this method can produce a high yield of nanovesicles, the selection of nano-sized structures solely on this basis presents significant disadvantages such the inability to remove similar exosomes of equal weight;^10^ deformations and damage associated with the centrifugation process, such as exosomal aggregation,^11^ which can potentially impact proteomic and RNA content analysis^12,13^ as well as inconsistencies related to using the same protocol with different rotors.^14^ Moreover, subsequent characterisation techniques used to confirm the nature of exosomes isolated through differential ultracentrifugation, such as visualisation using TEM, which has been traditionally used to observe these structures at high resolution, is not always able to confirm the nature of vesicles due to artefacts associated with the sample preparation process for TEM, which causes dehydration and collapse of vesicles;^8,12^ and the presence of matrix vesicles lacking a mineral phase.^5,15,16^ Therefore, methods which can select these vesicles based on consistently described markers in their composition are more reliable and appropriate indicators of their presence in the mineralising matrix and for further description of their biological role. Markers involved in ossification, abundantly present on the surface of these matrix vesicles, such as tissue non-specific alkaline phosphatase (TNAP)^4,17–19^ can be targeted and used for immuno-isolation using standard immunoprecipitation protocols.^20,21^ Although a much smaller population can be selected using this procedure, the presence of ossification markers on nano-sized vesicles can provide confirmation on their actual identity as well as involvement in the biomineralization process.

Extracting these vesicles from complex structures such as bone is very difficult if not impossible. *In vitro*, this technique can be a straightforward process, given the high number of osteoblastic cells that can be cultured in 2D. However, the lack of a three-dimensional environment and of a bone-like surrounding matrix may influence the physiological relevance of the collected vesicles. 3D cultures of osteoblastic cells can provide an ideal alternative to generate a rich source of vesicles which can be collected for analysis. Recently, we have developed an organotypic model of mature bone,^22^ which allows long-term culture of bone tissue, in excess of 1 year, and which shows a biochemical composition and structural organisation similar to mature bone at the centimetre scale. We present here a method for small-scale isolation of matrix vesicles from the growth medium of this bone-like system, based on surface marker immunoselection, which can provide a valuable tool for understanding the biological function of these structures.

## Aims and objectives

The goal of this work was to isolate a small sub-population of matrix vesicles from the total secretome of cells embedded in the organotypic bone system that we have recently developed. The work used immunoselection, combined with a panel of characterisation techniques in order to determine the role of these nanostructures in biological processes taking place in constructs.

## Materials and methods

### Development of the organotypic culture system

Constructs were developed using the methods described previously,^22^ by seeding fibrin hydrogels with an osteoblastic cell population derived from the periosteal tissue of femoral bones. The fibrin scaffolds were produced through the interaction of the normal plasma components, thrombin and fibrinogen. Bovine-derived thrombin powder (Calbiochem, EDM Chemicals; 1KU) was reconstituted using 0.1% w/v BSA and 5 ml F12K Nutrient Mixture (1×) with Kaighn's modification (Gibco Life Technologies) to make a final concentration of 200 U ml^−1^. Powdered bovine fibrinogen (Sigma Life Sciences) was reconstituted in F12K Nutrient Mixture (1×) with Kaighn's modification (Gibco Life Technologies) at a ratio of 20 mg ml^−1^. Thrombin was added to a solution containing the cell culture medium (either DMEM or αMEM) at a ratio of 50 μl ml^−1^ solution. The anti-fibrinolytic agents aminohexanoic acid (200 mM) and aprotinin (10 mg ml^−1^) were added to the thrombin solution at a ratio of 2 μl ml^−1^ in order to reduce the degradation rate of the fibrin gel, in order for it to match the rate of new matrix formation so that the mechanical integrity of the tissue can be maintained over longer periods of time. Hydrogels were generated by mixing 500 μl thrombin solution with 200 μl fibrinogen. Gels were allowed to polymerize for 30–40 minutes. Cells were seeded into the fibrin constructs immediately following gel polymerization; at a density of 100 K/2 ml of cell culture medium.

These cell-seeded gels were formed on a mineral backbone structure in order to encourage self-organisation of the hydrogels. These structures were composed of 2 calcium phosphate anchors, which were provided as retention points around which cells organised and contracted the fibrin gels over 7 days, producing three-dimensional constructs. The anchors also supplied a source of calcium and phosphorus which was required for the formation of bone. Anchors were created by mixing 2.5 g of β-TCP powder (<125 μm particle size) per 1 ml of orthophosphoric acid (3.5 M) to generate a paste, composed of a mixture of brushite and β-TCP. The liquid mixture was poured into individual, pre-shaped wells of moulds, placed on top of a shaking platform, to encourage uniform setting inside the shapes. 1.4 cm stainless steel insect pins (Austerlitz minutiens, Fine Science Tools, USA), with a diameter of 0.20 mm were inserted into the individual wells on the moulds before the mixtures advanced into a solid state. The mixtures containing the pins were allowed to fully harden for 3–4 hours and were sterilized overnight using UV light exposure, as well as with 70% EtOH for 30 minutes on the day of use. The final anchors had a trapezoidal shape and measured approximately 3 mm × 4 mm × 4 mm in height.

Following 1 month of culture, constructs were supplemented with full osteogenic DMEM culture medium containing β-glycerophosphate (10 mM), ascorbic acid (0.1 mM) and dexamethasone (10 nM) (Sigma Aldrich, Germany), to encourage further matrix deposition.

### Visual analysis of the structural evolution

The progress of ossification was monitored using micro-computed tomography. Acquisition settings consisted of an X-ray voltage of 50 kV and a tube current of 100 μA; an image pixel size of 13 μm, with an exposure time of 510 ms, a rotation step of 0.4 degrees and a frame averaging value of 2. Flat field correction was performed for image clarity and no further corrective functions were applied. Transfer functions were created in the CTVox software that allowed segmentation of the high-density matrix components, as well as for creating colour-coded versions of the components in the constructs. The same transfer function was used for all constructs in the same group of investigation.

### Collection of medium for vesicle isolation

Medium was collected from the culture wells at various time points and a volume of 1 ml was used for every isolation and characterisation procedure using the procedures described below.

### Development of the immuno-isolation system

Matrix vesicles were isolated from the culture medium of constructs by immunoprecipitation using 2.8 μm superparamagnetic Dynabeads (Invitrogen, Thermo Fisher Scientific, CA, USA), covalently coupled to protein G on their surface (approx. 17 kDa). Rabbit monoclonal antibodies against Rat Alkaline Phosphatase, Tissue Non-Specific (TNAP) were used to isolate the vesicles (Abcam, Cambridge, United Kingdom). Antibodies were attached to the magnetic beads through their F_c_ region during a 15 minutes incubation with rotation at room temperature, at a ratio of 5 μg of Ab/200 μl PBS containing 0.01% Tween-20 and 0.09% sodium azide, in which 1.5 mg magnetic beads were resuspended (Invitrogen, Thermo Fisher Scientific, CA, USA). The complex formed was washed by resuspending in 200 μl buffer and 1000 μl of culture medium containing the matrix vesicles was added to the formed Dynabeads–Ab complex. Samples and complex were incubated for 15 minutes at room temperature, with rotation. Following vesicle binding to the antibody, the beads–Ab–vesicle complex formed was washed four times using PBS washing buffer (Invitrogen, Thermo Fisher Scientific, CA, USA). The Ab–vesicle complex was eluted from the beads though the addition of 20 μl elution buffer (Invitrogen, Thermo Fisher Scientific, CA, USA). For gel electrophoresis, vesicles were resuspended in a loading mixture containing 2.5 μl NuPAGE LDS sample buffer (4×), 1 μl NuPAGE Reducing Agent (10×) (all from Thermo Fisher Scientific, CA, USA) and 6.5 μl dH_2_O and were incubated for 10 minutes at 70 °C. Samples were loaded onto NuPAGE Novex 4–12% Bis-Tris protein gels (1 mm thick) and were run at 160 V for 60 minutes using MOPS buffer. 500 μl NuPage Antioxidant (Thermo Fisher Scientific, CA, USA) was added to the buffer to maintain the reduced state of proteins during protein gel electrophoresis. A Novex Sharp Pre-stained protein standard was used for reference (Thermo Fisher Scientific, CA, USA). For controls, PBS was used instead of culture medium and samples were subjected to the same isolation procedure and steps.

### Analysis of size distribution

Dynamic light scattering (DLS) using a Zetasizer Nano ZS Instrument (Malvern Instruments Ltd, Malvern, United Kingdom) was used to characterize the culture medium of young (14 days) and mature (1 year) constructs at the nano-scale in order to detect the presence of extracellular vesicles. 2.5 ml of supplemented medium for cell culture and culture medium from young and mature constructs were diluted 1 : 10 in PBS, inserted into 12 mm polystyrene cuvettes (Malvern Instruments Ltd, Malvern, United Kingdom) and size measurements were taken at a 173 degrees scattering angle, using a RI parameter of 1.33, at 22 °C. A 4 mW, 633 laser was passed through the samples. 3 readings were taken per sample and averaged to obtain the size distribution of nanoparticles based on scattered light intensity.

### Nanoparticle tracking analysis

Nanoparticle tracking analysis (NTA) was performed using a NanoSight LM10 instrument (Malvern Instruments Ltd, Malvern, United Kingdom), in order to measure matrix vesicle size and concentration within the culture medium of constructs. Calibration of the machine was performed using 200 nm polystyrene latex microspheres. Culture medium samples from young (14 days) and old constructs (1 year) were collected and stored at −80 °C. Matrix vesicles were isolated using immunoprecipitation and used immediately for NTA.

Approximately 300 μl of nanovesicles suspended in buffer were injected into the NanoSight sample chamber. Control medium samples were diluted 1 : 10 in PBS prior to injection. In-between recordings, the loading chamber was washed with 0.2 μm-filtered PBS and dH_2_O for three times.

The particle detection threshold was set to 8 and camera gain was set to a value of 1. For each sample, two NTA videos of 60 seconds duration, containing recordings of the nanovesicles moving under Brownian motion, were collected using the NTA 2.2 software. Nanovesicles were tracked individually by the software based on scattered light when exposed to a 50 μm wide laser. The long-distance scattered light from the vesicles was detected by a 20× magnification microscope, connected to the recording camera. The matrix vesicles hydrodynamic diameters were calculated using the Stokes–Einstein equation and concentration was calculated based on volume. Data generated by the software concerning size and concentration was averaged to produce the final distribution for each sample.

### ATP detection

Mineralisation is an ATP-dependent process and staining for ATP was performed directly on the beads to detect its presence using quinacrine dihydrochloride.^23,24^ Quinacrine dihydrochloride ≥90%, produced by Bayer (Sigma-Aldrich, Germany), was reconstituted using deionised H_2_O (dH_2_O) and was used at a concentration of 10 μM, based on previous research.^25^ Following isolation, 750 μl of the bead–antibody–vesicle complexes and controls (beads–antibody complexes, where dH_2_O was used instead of culture medium) were incubated with the dye for 30 minutes at room temperature. Following incubation, the beads were washed four times with dH_2_O and samples were imaged using an Olympus Fluoview FV1000 confocal laser scanning microscope (Olympus, Tokyo, Japan) equipped with a multi-line argon laser FV5-LAMAR/LAMAR-2 and a Helium–Neon Green Laser FV5-LAHEG-2/FV5-LAHEG. Images acquired from excitation at a wavelength of 405 nm were collected using the Fluoview FV10-ASW software, version 4.2 (Olympus, Tokyo, Japan) with an exposure time of 20 μs per pixel.

### Affinity to collagen gels

The ability of these vesicles to interact with bone matrix was assessed through incubation on flat culture wells coated with collagen I, similar to the major organic component of bone. Matrix vesicles bound to the monoclonal antibody against TNAP were eluted from the beads using the procedures described above using 20 μl elution buffer (Thermo Fisher Scientific, USA) and were added to 300 μl TBS buffer. 100 μl of the re-suspended vesicles were applied in triplicate to 1.9 cm^2^ wells in 24 well plates coated with collagen I (Gibco, Life Technologies, Thermo Fisher Scientific, USA) to determine whether the vesicles would be able to bind to collagen type I. Antibody–vesicle complexes were incubated in the plates for 30 minutes at room temperature, followed by a further incubation step at 37 °C for 30 minutes to partially simulate the physiological context. To be able to detect the amount of binding, a secondary antibody against the rabbit monoclonal primary antibody was used. Following incubation, wells were washed three times with TBS to remove the unbound vesicles. Secondary donkey anti-rabbit antibodies conjugated to Alexa Fluor 488 fluorophores, were diluted to 10 μg ml^−1^ in TBS containing 1% BSA and 100 μl were applied in triplicate to the bottom of the wells and left to incubate for 1 hour at room temperature. Following incubation, tissue sections were washed three times with TBS for 1 minute. In order to check for non-specific antibody binding, the steps listed above were performed on additional wells, in triplicate without the addition of the primary antibodies. Samples were imaged using the confocal system described above, using a 10× objective lens. Images were acquired from excitation at a wavelength of 488 nm, in triplicate, for the samples, controls and antibody controls. Data was collected using the Fluoview FV10-ASW software, version 4.2 (Olympus, Tokyo, Japan).

### Statistical analysis

All data is presented as mean ± SD. Statistical analysis was performed using a one-tailed distribution *t*-test, with a heteroscedastic variance assumed. A *p* value lower than 0.01 or 0.05 was chosen for determining significance (MS Excel, Washington, USA).

## Results and discussion

The outer membranes of matrix nanovesicles are rich in tissue non-specific alkaline phosphatase (TNAP), a form of ALP involved in both skeletal mineralization and ectopic calcifications.^5,6,17,18,26^ TNAP is central to the ossification process, and on the MV membrane, it hydrolyses both ATP and the mineral inhibitor pyrophosphate (PPi) to monophosphate ions in order to aid in building the bone mineral. Deactivating defects in the TNAP gene in mice show matrix vesicles which do contain a mineral phase but are unable to self-nucleate and generate further crystal growth due to high-amounts of pyrophosphate, leading to symptoms similar to the hereditary condition hypophosphatasia,^4,18,19,27^ as well as rickets, osteomalacia and spontaneous fractures.

**Fig. 1:**
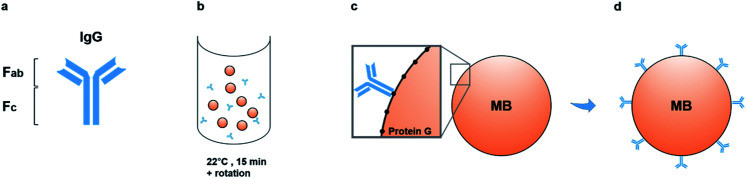
Development of the immuno-isolation method. Monoclonal IgG antibodies (a) were incubated with the magnetic beads for 15 minutes at room temperature with gentle rotation (b). During this incubation period, the antibodies bound *via* their F_c_ region to protein G, covalently coupled to the surface of the beads (c). Each bead recruited multiple antibodies, which were capable of binding the antigen of interest (TNAP) *via* their F_ab_ regions (d). MB = Magnetic Bead.

As such, the membrane-associated TNAP was targeted for immuno-capturing mineralisation nanovesicles and a schematic of the isolation system used to purify populations of nanostructures with similar characteristics to matrix vesicles is presented in Fig. 1. The method involved building an isolation complex by coupling TNAP monoclonal IgG antibodies (Ab) to recombinant protein G (17 kDa), which was covalently bound to the surface of 2.8 μm superparamagnetic beads (MBs). During a 15 minutes incubation at room temperature in PBS-Tween 20, the antibodies became bound *via* their F_c_ regions to the beads, leaving the F_ab_ regions on the antibodies available to bind TNAP on the outer surface of vesicles. A volume of 1.5 mg of magnetic beads was used, with the potential to recruit between 12.5–15 μg TNAP IgG. This volume was used throughout the whole procedure and recovered after each incubation step using magnetic isolation.

**Fig. 2:**
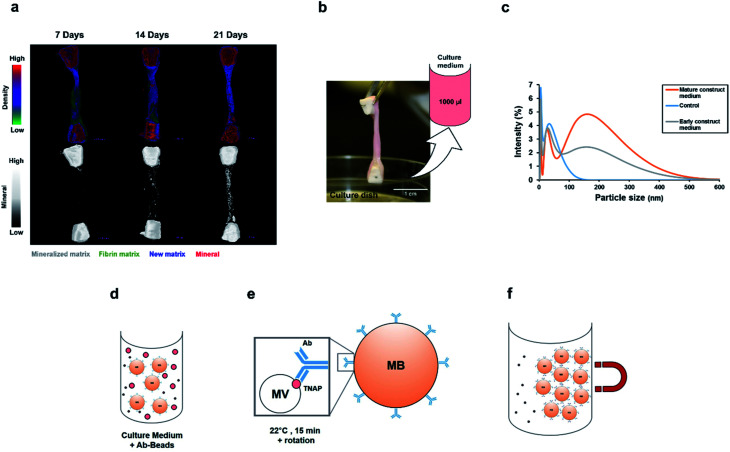
Immuno-isolation of matrix vesicles from organotypic bone culture medium. (a) Bone-tissue-like constructs containing osteoblastic cells were developed and cultured for extended periods of time, during which, a process of ossification took place and the matrix increased in density due to significant mineral deposition. Culture medium was collected from culture dishes at various time points (b) and subjected to nanoparticle analysis. (c) Dynamic light scattering analysis of samples from control (serum supplemented, cell-free), 14 days and 1 year-old medium samples. Compared with controls, media from both young and mature constructs contained nanoparticles in the size range of 50–300 nm. (d) Immuno-isolation of matrix vesicles from this population was performed by incubating 1000 μl of culture medium with the Ab–MB complexes for 15 minutes at room temperature, in PBS-Tween 20. Monoclonal antibodies attached to the vesicles containing TNAP on their outer membranes *via* their F_ab_ regions (e). The Ab–MB–MV complexes were separated from unbound antibodies and particles using magnetic separation (f). Ab = Antibody, MB = Magnetic Bead.

Organotypic bone constructs were developed by embedding periosteal osteoblastic cells into mechanically-strained fibrin scaffolds,^22^ which were cultured for extended periods of time, during which, the matrix became gradually replaced with a cell-produced greater density matrix containing increasing amounts of mineral (Fig. 2a). As the matrix underwent ossification, growth medium was collected from the culture wells (Fig. 2b) for extracting vesicles involved in crystal nucleation and mineralisation.

Before proceeding with vesicle immuno-isolation, secretome analysis using Dynamic Light Scattering (DLS) was performed on culture medium acquired from 14 days and 1 year-old constructs and controls to compare the normal distribution of nanoparticles at early and mature time points and with respect to normal supplemented medium (Fig. 2c). Interestingly, both the early and mature culture medium contained nanoparticles in the size range of 50 nm to 300 nm compared to controls (serum supplemented but cell-free medium), which encompasses the range in which previously described matrix mineralization vesicles were detected.^3,6,28^ Mature constructs (1 year), which have been previously shown to contain approximately 70% mineral,^22^ contained a higher concentration of these nanoparticles in their growth medium.

To determine whether this set of cell-secreted nanoparticles contained a sub-population of matrix vesicles involved in skeletal mineralization, the TNAP immuno-isolation procedure was used to separate this subset of vesicles from the rest of the secretome. The isolation procedure involved incubating 1000 μl of culture medium with the Ab–MB complex (Fig. 2d) for 15 minutes at room temperature, in PBS-Tween-20. During this incubation, the Ab–MB complexes attached to the surface of vesicles *via* F_ab_-TNAP binding (Fig. 2e). The remaining debris and unbound vesicles were removed by magnetic separation (2f). The Ab–MV complex was subsequently eluted from the beads and used with different analytical techniques.

**Fig. 3:**
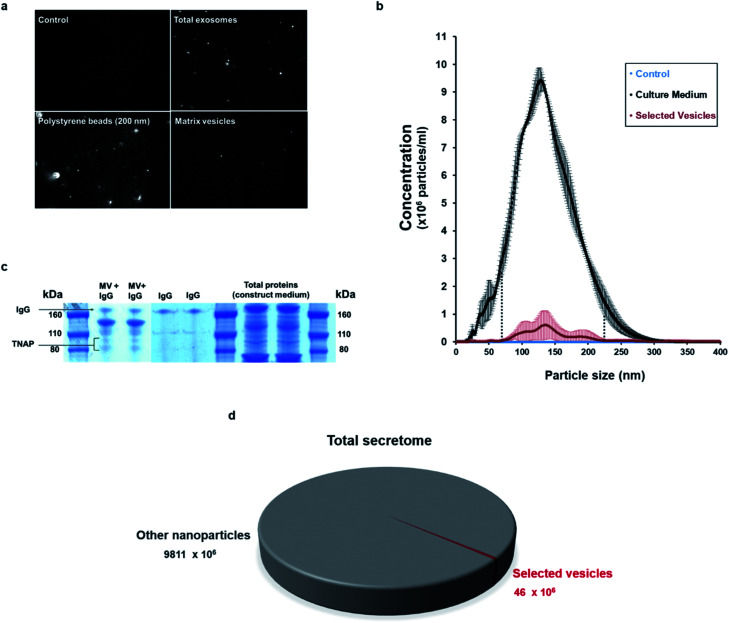
Characterisation of the immuno-selected vesicles. (a) Nanovesicles, visualised in real-time exclusively using NTA. Controls (sterile PBS) did not contain any nanoparticles. The growth medium of constructs contained an abundance of nanovesicles, whereas the buffer containing the isolated samples contained a smaller population of vesicles. Positive controls (200 nm polystyrene beads) are provided as reference. (b) The small population of purified vesicles was detected in a much narrower range than the total exosomes in the construct medium (dotted lines). Vesicles ranging between 100–200 nm were particularly abundant. Data is presented as average of multiple runs ± SD. *n*_matrix vesicles_ = 3; *n*_control_ = 3, *n*_total exosomes_ = 2. (c) When denatured using SDS-PAGE, the Ab–MV complexes separated into several fractions, which include the TNAP protein dimer (80–110 kDa), the IgG antibody (170 kDa) and several unidentified protein fractions (110–160 kDa). (d) The TNAP-containing population of vesicles selected accounts for approximately 0.005% of the total secretome.

Nanoparticle tracking analysis (NTA) performed on the isolated potential matrix vesicles as well as non-purified construct medium samples (Fig. 3a and b), indicated that it was possible to isolate a small population of TNAP-containing nanostructures with sizes in a much narrower range than the ones present in the growth medium (Fig. 3b) and compared to similar studies conducted using traditional ultracentrifugation methods.^9,29^ The highest concentrations were those of particles which ranged in size from 100–200 nm (Fig. 3b). This corresponds to the range in which previously described matrix mineralization vesicles were detected by previous authors.^3,5,6,26^

When denatured under SDS-gel electrophoresis, MV–IgG complexes eluted from the beads separated into several fractions (Fig. 3c). The TNAP band can be observed as a dimer between 80–110 kDa,^30,31^ and IgG bands appeared at around 170 kDa, as confirmed by antibody controls. Fractions of other, unidentified proteins appeared between 110–160 kDa, further supporting the proteomic complexity of the isolated structures, as previously reported with MVs.

**Fig. 4:**
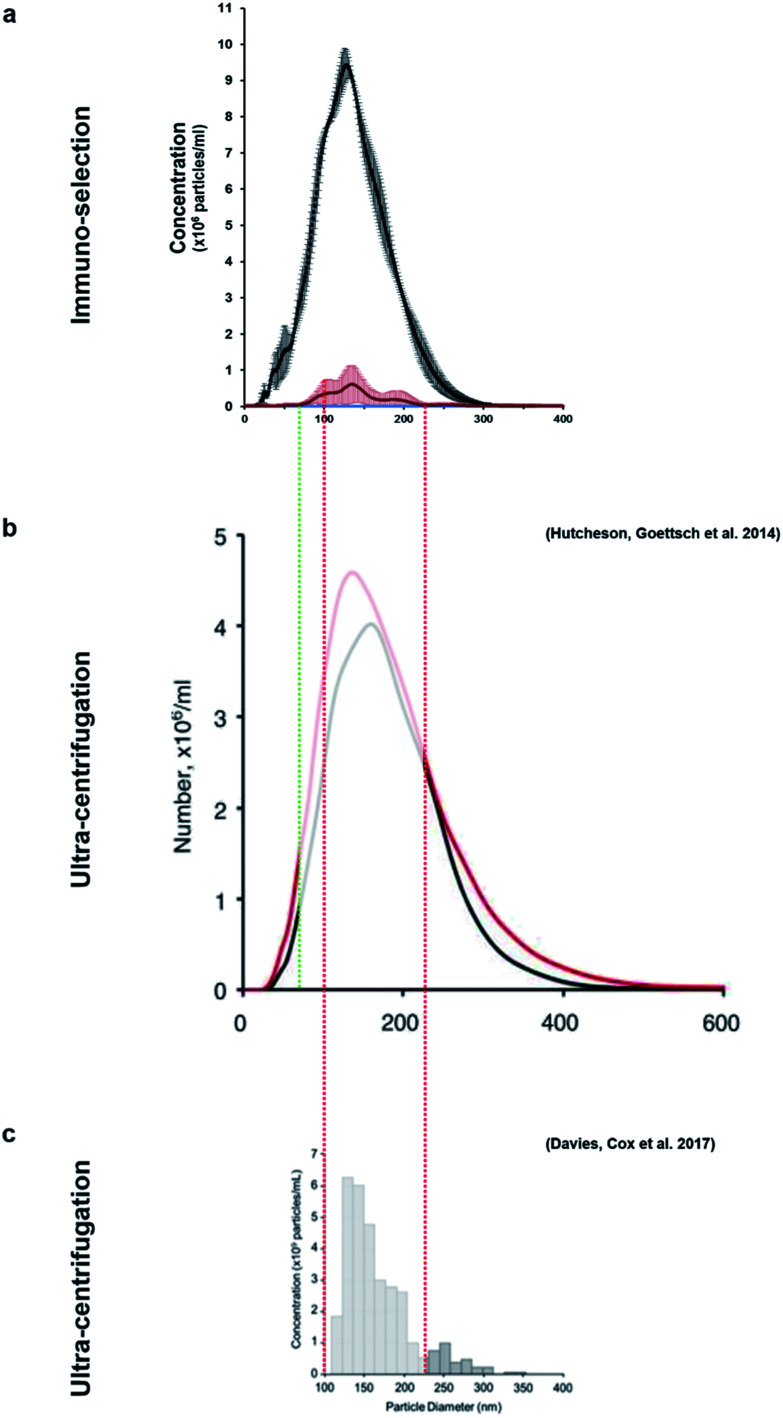
The size-variable concentrations of mineralisation vesicles obtained (a) from immuno-isolation in this study compared to extensive studies in which these vesicles were isolated from culture using different ultracentrifugation protocols (b and c). (b)^9^ presents the size distribution of vesicles from mineralising (red) and non-mineralising controls (black) and image (c)^29^ presents mineralising samples only. This study (a, red) shows that the vesicles isolated using immuno-selection belong to a much narrower size range (predominantly 100–200 nm, red/green dotted lines), considerably reducing the amount of potential contaminating exosomes of similar size. Images (b) and (c) are used under the terms of the Creative Commons Attribution Licence (CC-BY-NC 3.0 Unported and CC-BY respectively).

This method allowed the isolation of approximately 46 million TNAP containing vesicles per 1 ml of bone culture medium, accounting for approximately 0.005% of the total secretome (total cell products recorded in the same amount) (Fig. 3d). The successful separation of this small population from the medium of constructs, which contains a significantly high amount of proteins (Fig. 3c) is an advantage over traditional ultracentrifugation protocols (Fig. 4a–c), where rotation at high velocities can result in protein aggregation, which can lead to false positive results.^32,33^

**Fig. 5:**
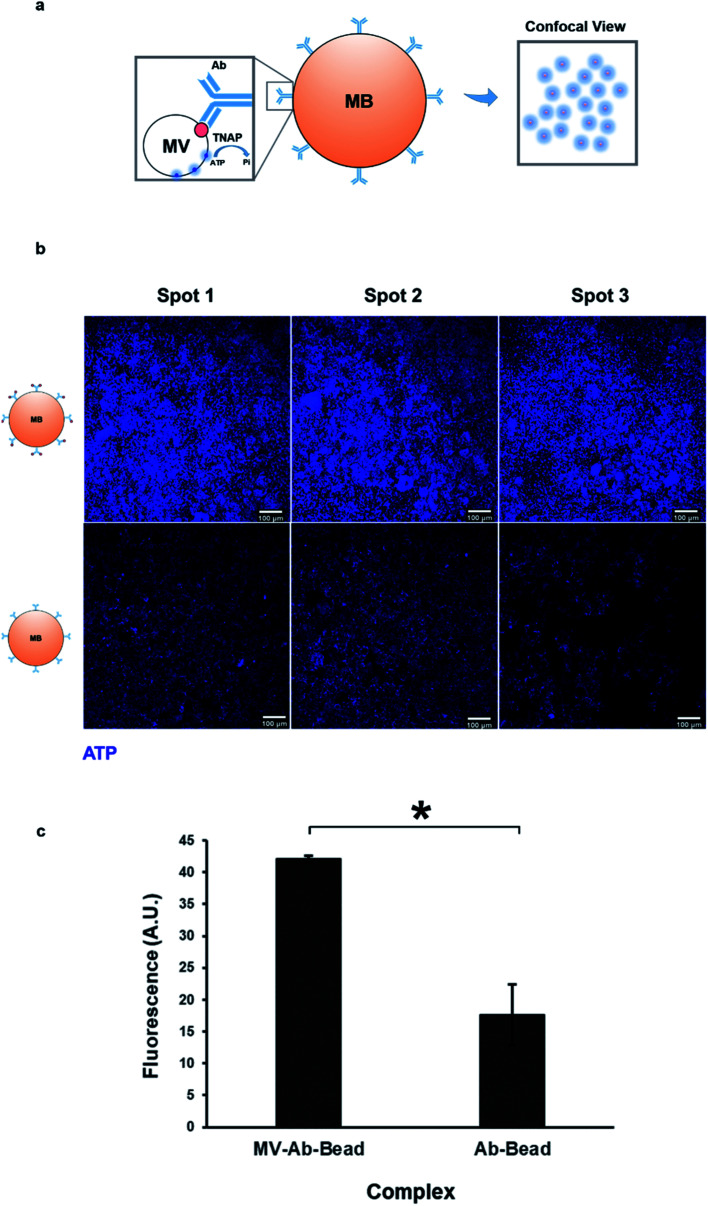
Isolated matrix vesicles contain ATP. (a) Mineralisation is an ATP-mediated process. ATP is used by membrane-bound TNAP on the surface of vesicles to generate Pi. Vesicular ATP was labelled using quinacrine dihydrochloride and visualised indirectly using confocal microscopy, where it was detected as blue fluorescence surrounding the beads. (b) Purified MVs and controls were stained directly on the magnetic beads using quinacrine dihydrochloride. Controls contained the bound antibody but were treated with dH_2_O as opposed to culture medium. All images were acquired under the same conditions and settings. Some degree of background staining can be observed in control beads. The vesicles-bound beads generate a significantly higher amount of fluorescence (c, *p < 0.01), indicating that the presence of ATP is associated with the MVs.

As mentioned previously, TNAP is the major phosphatase of MV vesicles, which regulates the generation of extracellular Pi, and this is an ATP-mediated process.^5,18^ These vesicles can maximally initiate the deposition of calcium in the presence of this organic chemical.^34^ The presence of ATP in matrix vesicles was investigated using quinacrine dihydrochloride staining and was imaged indirectly using confocal microscopy of bead complexes (Fig. 5a). The beads which were bound to the selected matrix vesicles generated a significantly higher amount of fluorescence compared to controls (beads bound to the antibody-only) (Fig. 5b and c). The detection of ATP co-localised with the vesicles could be indicative of an active role of these structures in the mineralisation process of constructs.

The role of these vesicles was probed further by assessing their osteogenic potential, or their ability to bind the bone extracellular matrix. Matrix vesicles have been shown to bind to collagen type I, II and X^35^ and collagen I has been shown to induce the vesicle-mediated mineralisation of articular cartilage embedded in agarose–collagen hydrogels.^36^ The affinity of the isolated vesicles to collagen I was tested by incubating the eluted vesicle–antibody complexes from the beads to flat, collagen I-coated wells during a 1 hour incubation regime.

**Fig. 6:**
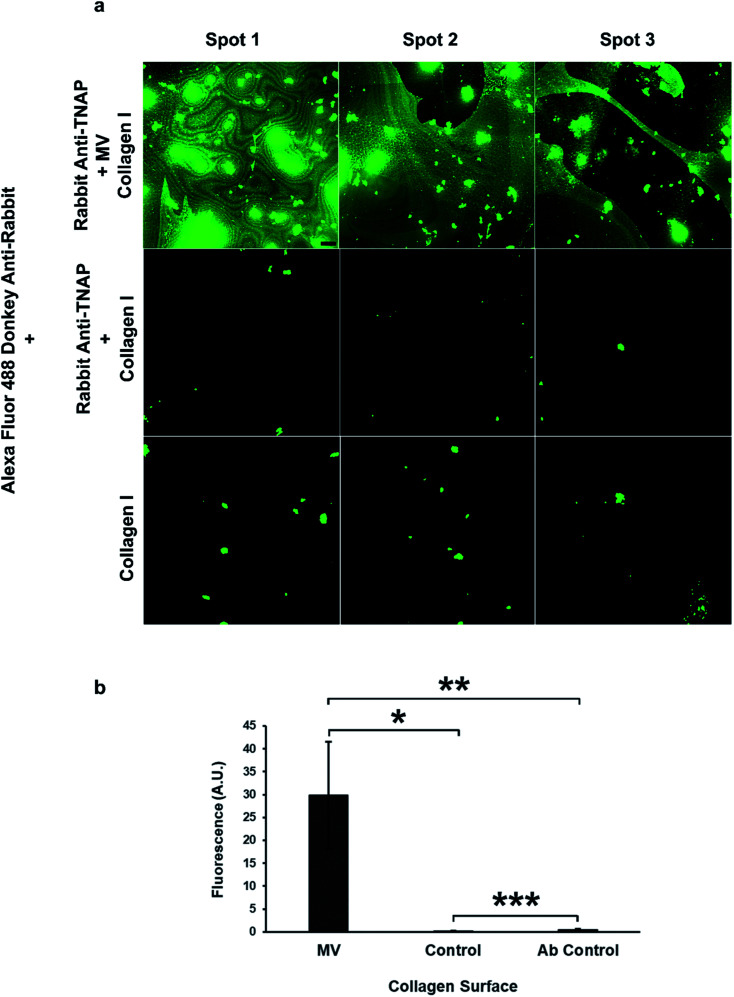
Isolated matrix vesicles show a high degree of osteocompetency. (a) The TNAP Ab-bound vesicles have the ability to significantly bind collagen type I (top row, (b) *p < 0.05), compared to controls, which contained eluted TNAP antibodies only and showed a small degree of non-specific binding (centre). Collagen binding was detected using Alexa Fluor 488 conjugated secondary antibodies against the primary rabbit TNAP Ab. These secondary antibodies showed a small degree of non-specific binding to the collagen I surfaces ((a) bottom row). The fluorescence of samples containing MVs was significantly higher than the Ab control samples ((b) **p < 0.05). Images were acquired using the same settings and conditions. Scale bar = 100 μm; ***p < 0.05.

The amount of binding of the matrix vesicles to the collagen surfaces was detected by applying a fluorescently-tagged (Alexa Fluor 488 conjugated) secondary antibody against the primary TNAP antibodies that remained bound to these structures. Results are presented in Fig. 6a and b and show that MV-incubated collagen surfaces generated significantly higher amounts of fluorescence compared to controls, which contained the primary antibody-only. Both the primary and the secondary antibodies showed a small degree of non-specific binding, however, surfaces which were bound to matrix vesicles generated significantly higher fluorescence in all cases. These results suggest that purified matrix vesicles have a high degree of affinity to the collagen matrix and are therefore osteocompetent.

## Conclusions

This work presented a method for TNAP-dependent immunocapture of specific matrix vesicles involved in ossification from organotypic bone culture systems, thus providing a new source of these inaccessible structures. A small, distinct population of TNAP containing vesicles can be isolated using this method and these structures appear to contain ATP and have an affinity to collagen I, suggesting a high degree of osteocompetency and that the population selected is representative of the *in vivo* structures.

This isolation procedure can undergo further optimisation by increasing the incubation times of vesicles with the antibody-bound beads and by applying a range of physiologically-relevant temperature intervals.

A higher yield could be generated by using purification beads of larger size, as the bigger volume would allow higher amounts of protein G on the surface, therefore IgG recruitment and hence available binding slots for selecting matrix vesicles.

Vesicles recovered from this method can be used in further applications that could clarify their involvement in the mineralisation process. This includes chemical characterisation of protein content using mass spectrometry, for detecting annexins^2,5,6,11^ and peptidases commonly described as being part of matrix vesicles.^37–39^ Fluorescence imaging using super high-resolution microscopy (SIM, STED, PALM, STORM)^40,41^ and TEM^2,4^ could allow visualisation of these nanostructures.

Demonstration of their role in cartilage mineralisation could also be attempted using collagen type II–X surfaces.^35^ Several molecular techniques (qPCR, microarrays, ELISA, Western Blots) could allow detection and isolation of MMPs, integrins, transport proteins and miRNA species, which could be used to characterise the molecular role of these structures.^42–44^

## Conflicts of interest

There are no conflicts of interest to declare.

## Supplementary Material

## References

[cit1] Mackie E. J., Ahmed Y. A., Tatarczuch L., Chen K. S., Mirams M. (2008). Endochondral ossification: how cartilage is converted into bone in the developing skeleton. Int. J. Biochem. Cell Biol..

[cit2] Anderson H. C. (2003). Matrix vesicles and calcification. Curr. Rheumatol. Rep..

[cit3] Anderson H. C. (1969). Vesicles associated with calcification in the matrix of epiphyseal cartilage. J. Cell Biol..

[cit4] Anderson H. C., Sipe J. B., Hessle L., Dhanyamraju R., Atti E., Camacho N. P. (2004). *et al.*, Impaired calcification around matrix vesicles of growth plate and bone in alkaline phosphatase-deficient mice. Am. J. Pathol..

[cit5] Cui L., Houston D. A., Farquharson C., MacRae V. E. (2016). Characterisation of matrix vesicles in skeletal and soft tissue mineralisation. Bone.

[cit6] Golub E. E. (2009). Role of Matrix Vesicles in Biomineralization. Biochim. Biophys. Acta.

[cit7] Felix R., Herrmann W., Fleisch H. (1978). Stimulation of precipitation of calcium phosphate by matrix vesicles. Biochem. J..

[cit8] Li P., Kaslan M., Lee S. H., Yao J., Gao Z. (2017). Progress in Exosome Isolation Techniques. Theranostics.

[cit9] Hutcheson J. D., Goettsch C., Pham T., Iwashita M., Aikawa M., Singh S. A. (2014). *et al.*, Enrichment of calcifying extracellular vesicles using density-based ultracentrifugation protocol. J. Extracell. Vesicles.

[cit10] Safdar A., Saleem A., Tarnopolsky M. A. (2016). The potential of endurance exercise-derived exosomes to treat metabolic diseases. Nat. Rev. Endocrinol..

[cit11] Chiou N.-T., Ansel K. M. (2016). Improved exosome isolation by sucrose gradient fractionation of ultracentrifuged crude exosome pellets. Nature Exchange Protocols.

[cit12] Lobb R. J., Becker M., Wen S. W., Wong C. S., Wiegmans A. P., Leimgruber A. (2015). *et al.*, Optimized exosome isolation protocol for cell culture supernatant and human plasma. J. Extracell. Vesicles.

[cit13] Lamparski H. G., Metha-Damani A., Yao J. Y., Patel S., Hsu D. H., Ruegg C. (2002). *et al.*, Production and characterization of clinical grade exosomes derived from dendritic cells. J. Immunol. Methods.

[cit14] Livshits M. A., Khomyakova E., Evtushenko E. G., Lazarev V. N., Kulemin N. A., Semina S. E. (2015). *et al.*, Isolation of exosomes by differential centrifugation: theoretical analysis of a commonly used protocol. Sci. Rep..

[cit15] Landis W. J., Paine M. C., Glimcher M. J. (1977). Electron microscopic observations of bone tissue prepared anhydrously in organic solvents. J. Ultrastruct. Res..

[cit16] Landis W. J., Glimcher M. J. (1982). Electron optical and analytical observations of rat growth plate cartilage prepared by ultracryomicrotomy: the failure to detect a mineral phase in matrix vesicles and the identification of heterodispersed particles as the initial solid phase of calcium phosphate deposited in the extracellular matrix. J. Ultrastruct. Res..

[cit17] Ciancaglini P., Simao A. M., Camolezi F. L., Millan J. L., Pizauro J. M. (2006). Contribution of matrix vesicles and alkaline phosphatase to ectopic bone formation. Braz. J. Med. Biol. Res..

[cit18] Hessle L., Johnson K. A., Anderson H. C., Narisawa S., Sali A., Goding J. W. (2002). *et al.*, Tissue-nonspecific alkaline phosphatase and plasma cell membrane glycoprotein-1 are central antagonistic regulators of bone mineralization. Proc. Natl. Acad. Sci. U. S. A..

[cit19] Millán J. L., Whyte M. P. (2016). Alkaline Phosphatase and Hypophosphatasia. Calcif. Tissue Int..

[cit20] Wittrup A., Zhang S.-H., Svensson K. J., Kucharzewska P., Johansson M. C., Mörgelin M. (2010). *et al.*, Magnetic nanoparticle-based isolation of endocytic vesicles reveals a role of the heat shock protein GRP75 in macromolecular delivery. Proc. Natl. Acad. Sci. U. S. A..

[cit21] Hussain S., Davanger S. (2015). Postsynaptic VAMP/Synaptobrevin Facilitates Differential Vesicle Trafficking of GluA1 and GluA2 AMPA Receptor Subunits. PLoS One.

[cit22] Iordachescu A., Amin H. D., Rankin S. M., Williams R. L., Yapp C., Bannerman A. *et al.*, An *In Vitro* Model for the Development of Mature Bone Containing an Osteocyte Network. Adv. Biosyst..

[cit23] Akopova I., Tatur S., Grygorczyk M., Luchowski R., Gryczynski I., Gryczynski Z. (2012). *et al.*, Imaging exocytosis of ATP-containing vesicles with TIRF microscopy in lung epithelial A549 cells. Purinergic Signalling.

[cit24] Orriss I. R., Knight G. E., Utting J. C., Taylor S. E. B., Burnstock G., Arnett T. R. (2009). Hypoxia stimulates vesicular ATP release from rat osteoblasts. J. Cell. Physiol..

[cit25] Loncar R., Zotz R. B., Sucker C., Vodovnik A., Mihalj M., Scharf R. E. (2007). Platelet adhesion onto immobilized fibrinogen under arterial and venous *in vitro* flow conditions does not significantly differ between men and women. Thromb. J..

[cit26] Golub E. E. (2011). Biomineralization and matrix vesicles in biology and pathology. Semin. Immunopathol..

[cit27] Mornet E. (2000). Hypophosphatasia: the mutations in the tissue-nonspecific alkaline phosphatase gene. Hum. Mutat..

[cit28] Bonucci E. , Biological Calcification: Normal and Pathological Processes in the Early Stages, Springer Berlin Heidelberg, 2007

[cit29] Davies O. G., Cox S. C., Williams R. L., Tsaroucha D., Dorrepaal R. M., Lewis M. P. (2017). *et al.*, Annexin-enriched osteoblast-derived vesicles act as an extracellular site of mineral nucleation within developing stem cell cultures. Sci. Rep..

[cit30] Satou Y., Al-Shawafi H. A., Sultana S., Makita S., Sohda M., Oda K. (2012). Disulfide bonds are critical for tissue-nonspecific alkaline phosphatase function revealed by analysis of mutant proteins bearing a C201-Y or C489-S substitution associated with severe hypophosphatasia. BBA, Biochim. Biophys. Acta, Mol. Basis Dis..

[cit31] Ishida Y., Komaru K., Oda K. (2011). Molecular characterization of tissue-nonspecific alkaline phosphatase with an Ala to Thr substitution at position 116 associated with dominantly inherited hypophosphatasia. BBA, Biochim. Biophys. Acta, Mol. Basis Dis..

[cit32] Berkowitz S. A. (2006). Role of analytical ultracentrifugation in assessing the aggregation of protein biopharmaceuticals. AAPS J..

[cit33] den Engelsman J., Garidel P., Smulders R., Koll H., Smith B., Bassarab S. (2011). *et al.*, Strategies for the Assessment of Protein Aggregates in Pharmaceutical Biotech Product Development. Pharm. Res..

[cit34] Hsu H. H., Anderson H. C. (1978). Calcification of isolated matrix vesicles and reconstituted vesicles from fetal bovine cartilage. Proc. Natl. Acad. Sci. U. S. A..

[cit35] Wu L. N., Genge B. R., Lloyd G. C., Wuthier R. E. (1991). Collagen-binding proteins in collagenase-released matrix vesicles from cartilage. Interaction between matrix vesicle proteins and different types of collagen. J. Biol. Chem..

[cit36] Jubeck B., Gohr C., Fahey M., Muth E., Matthews M., Mattson E. (2008). *et al.*, Promotion of articular cartilage matrix vesicle mineralization by type I collagen. Arthritis Rheum..

[cit37] Xiao Z., Camalier C. E., Nagashima K., Chan K. C., Lucas D. A., de la Cruz M. J. (2007). *et al.*, Analysis of the extracellular matrix vesicle proteome in mineralizing osteoblasts. J. Cell. Physiol..

[cit38] Xiao Z., Conrads T. P., Beck G. R., Veenstra T. D. (2008). Analysis of the extracellular matrix and secreted vesicle proteomes by mass spectrometry. Methods Mol. Biol..

[cit39] Hirschman A., Deutsch D., Hirschman M., Bab I. A., Sela J., Muhlrad A. (1983). Neutral peptidase activities in matrix vesicles from bovine fetal alveolar bone and dog osteosarcoma. Calcif. Tissue Int..

[cit40] Wegel E., Göhler A., Lagerholm B. C., Wainman A., Uphoff S., Kaufmann R. (2016). *et al.*, Imaging cellular structures in super-resolution with SIM, STED and localisation microscopy: a practical comparison. Sci. Rep..

[cit41] Huang B., Bates M., Zhuang X. (2009). Super resolution fluorescence microscopy. Annu. Rev. Biochem..

[cit42] Chaturvedi P., Chen N. X., O'Neill K., McClintick J. N., Moe S. M., Janga S. C. (2015). Differential miRNA Expression in Cells and Matrix Vesicles in Vascular Smooth Muscle Cells from Rats with Kidney Disease. PLoS One.

[cit43] Lin Z., Rodriguez N. E., Zhao J., Ramey A. N., Hyzy S. L., Boyan B. D. (2016). *et al.*, Selective enrichment of microRNAs in extracellular matrix vesicles produced by growth plate chondrocytes. Bone.

[cit44] Jiang L., Cui Y., Luan J., Zhou X., Zhou X., Han J. (2013). A comparative proteomics study on matrix vesicles of osteoblast-like Saos-2 and U2-OS cells. Intractable Rare Dis. Res..

